# Implications of Cellular Aging in Cardiac Reprogramming

**DOI:** 10.3389/fcvm.2018.00043

**Published:** 2018-04-27

**Authors:** Fabiana Passaro, Gianluca Testa

**Affiliations:** ^1^Department of Molecular Medicine and Medical Biotechnology, University of Naples “Federico II”, Napoli, Italy; ^2^Interdepartmental Center for Nanotechnology Research – NanoBem, University of Molise, Campobasso, Italy; ^3^Department of Medicine and Health Sciences “Vincenzo Tiberio”, University of Molise, Campobasso, Italy

**Keywords:** cellular senescence, SASP, reprogramming of somatic cells, cardiovascular aging, direct cardiac reprogramming

## Abstract

Aging is characterized by a chronic functional decline of organ systems which leads to tissue dysfunction over time, representing a risk factor for diseases development, including cardiovascular. The aging process occurring in the cardiovascular system involves heart and vessels at molecular and cellular level, with subsequent structural modifications and functional impairment. Several modifications involved in the aging process can be ascribed to cellular senescence, a biological response that limits the proliferation of damaged cells. In physiological conditions, the mechanism of cellular senescence is involved in regulation of tissue homeostasis, remodeling, and repair. However, in some conditions senescence-driven tissue repair may fail, leading to the tissue accumulation of senescent cells which in turn may contribute to tumor promotion, aging, and age-related diseases. Cellular reprogramming processes can reverse several age-associated cell features, such as telomere length, DNA methylation, histone modifications and cell-cycle arrest. As such, induced Pluripotent Stem Cells (iPSCs) can provide models of progeroid and physiologically aged cells to gain insight into the pathogenesis of such conditions, to drive the development of new therapies for premature aging and to further explore the possibility of rejuvenating aged cells. An emerging picture is that the tissue remodeling role of cellular senescence could also be crucial for the outcomes of *in vivo* reprogramming processes. Experimental evidence has demonstrated that, on one hand, senescence represents a cell-autonomous barrier for a cell candidate to reprogramming, but, on the other hand, it may positively sustain the reprogramming capability of surrounding cells to generate fully proficient tissues. This review fits into this conceptual framework by highlighting the most prominent concepts that characterize aging and reprogramming and discusses how the aging tissue might provide a favorable microenvironment for *in vivo* cardiac reprogramming.

## Introduction

In the last decades, developed countries have faced a significant rise in life expectancy with consistent dramatic increase of the elderly population (1).

Aging is the major risk factor for cardiovascular diseases, which are the leading cause of morbidity, disability, and death in western countries (2).

The aging process occurring in the cardiovascular system involves heart and vessels at molecular and cellular level with consequent structural modifications and functional impairment (3).

Cardiac aging involves on one hand, cellular macromolecular and mitochondrial and energetic changes and, on the other hand, cell renewal mechanisms and stem cell function. In addition, also extracellular matrix (ECM) alterations contribute to the structural changes that ultimately lead to cardiac dysfunction commonly observed in the elderly (4).

Several modifications involved in the process of cardiac aging can be ascribed to cellular senescence (3). A role for cellular senescence has been hypothesized in the development of cardiovascular diseases which are frequently associated with aging, like atherosclerosis and heart failure. The activation of the cellular senescence genetic program prompts a series of molecular changes, mostly affecting cell cycle, ECM, secretion of growth factors, and inflammatory mediators. Moreover, cellular senescence has been demonstrated crucial for the homeostasis of stem cell reservoir, thus pointing to its key role in tissue remodeling, both in physiological and pathological conditions (5).

In the attempt to regenerate functional cardiomyocytes (CMs), different approaches have been developed in recent decades, ranging from the stimulation of the intrinsic proliferative capacity of resident CMs to the enhancement of resident or not resident (tissue grafts) cardiac progenitor cells differentiation (6–9).

The discovery of induced Pluripotent Stem Cells (iPSCs) by Takahashi and Yamanaka in 2006 (10) has changed the field of cardiac regenerative medicine, unveiling a new approach to heart regeneration. Since then, either the cardiac differentiation of iPSCs or the conversion of one differentiated cell type into another, without proceeding through a pluripotent intermediate, the so-called “direct reprogramming”, was reported for different cell types including CMs (11).

### Reprogramming Processes in the Aging Contest

The manipulation of cell fates through reprogramming has deeply changed the established concepts about the stability of cellular identity, leading to new fields of research in human disease modeling, *in vitro* tissue differentiation and cellular trans-differentiation (12).

Since 2006, when Takahashi and Yamanaka (10) announced the successful derivation of iPSCs from adult mouse fibroblasts through the ectopic co-expression of the four genes OCT4, SOX2, KLF4 and c-MYC (OSKM), further studies reported of successful reprogramming of a wide variety of other human cell types (13–15). These results demonstrated the apparently universal capacity to alter cellular identity.

As iPSCs maintain the key features of ES cells, including the ability to give rise to any cell type within the body, this technology paved the way to innovative cell replacement therapies. However, transplantation of iPSC-derived cells raises several safety concerns. For instance, iPSC-derived cardiomyocytes frequently display different electrophysiological characteristics and immature functionality, making these cells unsuitable for transplantation (16).

In general, clinical application of this *in vitro* technology is a challenge, as *in vitro* manipulation of cells still brings up concerns on cell contamination and accumulation of mutations, transplantation procedures, delivery strategies and retention of the graft.

Regarding aging and age-related disorders, several experimental evidence demonstrated that, even though aging is a barrier to reprogramming, an aged cell may still be reprogrammed (17). Thus, iPSCs may provide models of progeroid and physiologically aged cells to gain insight into the pathogenesis of such conditions, to develop new therapies for premature aging and to further explore the possibility of rejuvenating aged cells.

Nevertheless, the latter may also represent a major problem of modeling aging with iPSCs as, once reprogrammed, aged cells no longer display age-associated characteristics such as telomere shortening, reduced mitochondrial fitness, and cellular senescence (18–21). This situation hampers the use of rejuvenating cells for the study of late-onset alterations, making it necessary to expose such cells to treatments inducing age- and stress-related conditions.

As aging constitutes a critical barrier to cell reprogramming, in aged cells it might be helpful to counteract some age-associated characteristics to increase reprogramming efficiency (18, 19, 22–24). Among age-related alterations, cellular senescence seems to negatively impact the reprogramming process (25).

### Cellular Senescence as a Tissue-Remodeling Mechanism

Cellular senescence was first described in 1961 by Hayflick and Moorhead (26) as a process that reduced the proliferation of normal human cells in culture. Interestingly, they also argued that senescence could have a pivotal role in driving the aging process *in vivo*. In the last decades, *in vitro* and *in vivo* evidence have contributed to defining senescence as an irreversible cell cycle arrest occurring when a proliferating cell is exposed to a severe genotoxic stress, thus revealing a multifaceted phenomenon combining both genetic and environmental components, which operate through convergent pathways (27, 28).

Today we know that this phenomenon described by Hayflick and Moorhead accounts for the so-called “replicative senescence” (RS), which refers to the irreversible cell cycle arrest due to the progressive telomeres attrition at each S phase (29), and that occurs after extensive proliferation in the absence of endogenous telomerase activity (30–32). Apart from embryonic stem cells (33), which express telomerase, in principle, all proliferating cells can undergo RS (27).

From a molecular point of view, cell senescence is believed to have evolved as a mechanism aimed at the prevention of the replication and transmission of damaged DNA to future generations of cells, thus playing a role in the orchestration of tissue remodeling and repair, and acting as a tumor suppressor mechanism (5). In this sense, it is not surprising that cellular senescence has been also implicated in embryonic development, both in mice and humans (34).

However, regardless of their inability to replicate, senescent cells are still metabolically active. In particular, they develop an aberrant gene expression profile leading to the over-expression of different proteins, mostly secreted and not expressed by the young counterpart, which confers a proinflammatory behavior, the so-called Senescence-Associated Secretory Phenotype (SASP), able to induce or accelerate changes in surrounding normal tissues (35).

In general, senescent cells are efficiently cleared by phagocytosis, but, in some conditions, tissue repair may fail, leading to senescent cells accumulation in tissues, which in turn may contribute to tumor promotion, tissue aging and age-related diseases (5, 27, 28).

The SASP comprises the secretion of chemokines, pro-inflammatory cytokines (with a particularly relevant role for interleukin-6 (IL-6) and interleukin-8 (IL-8)), growth factors and proteases (36–38), whose secretion triggers inflammation, which is critical for macrophages chemoattraction and the subsequent clearance of senescent cells by phagocytosis (39, 40). Nevertheless, SASP components may also induce senescence in surrounding cells in a paracrine manner, through a mechanism involving Reactive Oxygen Species (ROS) generation and DNA damage (41, 42). Recently, a role for SASP in inducing somatic stem/progenitor characteristics in several tissues has also been described (43).

Besides SASP, other peculiar characteristics of senescent cells are: the appearance of senescence-associated heterochromatin foci (SAHF), the expression of Senescence-Associated β-galactosidase (SA-β-gal), enlarged and flattened morphology, the presence of senescence-associated DNA-damage foci (SDFs) (44, 45).

Different stimuli, other than telomere erosion, may induce senescence (5). A common feature of such senescence-inducing stimuli is to elicit epigenomic disruption and genomic damage with the DNA damage response (DDR) activation, responsible for initiation and maintenance of both the *in vitro* and *in vivo* senescence growth arrest in mouse and human cells (46). These stimuli converge on activation of p53 and of cyclin-dependent kinase (CDK) inhibitors p16INK4A, p14ARF, p21CIP1 and p27 (47, 48). CDK–cyclin complexes inhibition leads to hypo-phosphorylation of the Retinoblastoma protein (Rb), with the subsequent proliferation arrest (49).

Other stimuli triggering persistent DDR signaling, such as oncogene-driven mitogenic signals or over-expression of pro-proliferative genes that cause defective replication origins and replication fork collapse, also leads to a senescence growth arrest, the so-called “oncogene-induced senescence” (OIS) (50–52). Also, treatments with cytotoxic chemotherapeutic agents, such as etoposide, or de-regulation of some microRNA that break DNA double strand, may be responsible for a p53-dependent premature senescence (53–55). Senescence can also occur, however, without detectable DDR signaling. This “stress-induced senescence” (SIS) could be dependent on several endogenous or exogenous sources of stress, such as ROS (56, 57).

More recently, the role of cellular senescence in tissue remodeling has been linked to cellular reprogramming processes (18).

It is well established that cell senescence causes a p53-dependent block to reprogramming (22–25). Indeed, improved reprogramming efficiency has been achieved by the knocking-down expression of p53, p21CIP1, p16INK4A and p14ARF (25, 58, 59), and microRNA-195 (60).

The transgenic expression of the four Yamanaka factors (OSKM) (10) in adult mice, in addition to reprogramming, also induces cellular damage and senescence, both *in vitro* (25) and *in vivo* (61, 62). These two opposite cellular fates are detectable in different subsets of cells, albeit in the same tissue (61, 62).

These results support the idea that cellular senescence may influence the outcome of cell-fate manipulating procedure in two ways: on the one hand it represents a cell-autonomous barrier for a cell candidate to reprogramming, but, on the other hand, it may positively sustain the reprogramming capability of surrounding cells to generate fully proficient tissues. Therefore, the emerging picture is that senescence, via SASPs, may generate a tissue microenvironment sustaining *in vivo* reprogramming (63).

### Cellular Senescence: A Cell-Autonomous Barrier for Reprogramming

The number of senescent cells increases in organs during aging (45, 48). Consequently, old age donor cells may contain a more significant number of senescent and pre-senescent cells, and these would ultimately affect the reprogramming efficiency.

Moreover, the reprogramming process itself triggers senescence, the so-called reprogramming-induced senescence (RIS) (25, 62). Cells from old donors may, therefore, be more sensitive to RIS and more difficult to reprogram, due to the already activated intrinsic senescence pathways.

Studies investigating the possible influence of senescence on reprogramming highlighted a negative association between replicative passages of cultured cells and reprogramming efficiency, with a prominent role of p16/p21-dependent senescence response in thwarting aging cell-fate manipulations (25), and, thus, confirming the complex role played by the Ink4a/Arf locus in reprogramming (58, 62).

Pioneer studies aimed at investigating the effect of donor age on reprogramming efficiency were performed in mice and revealed that the older the donor mouse, the lower the extent of reprogramming efficiency (64–66). Indeed, a significant reduction was observed in the reprogramming efficiency of aged vs. young mouse dermal fibroblasts or bone marrow cells, in which those from older animals showed either lower or slower reprogramming (64–66). Nevertheless, these studies also demonstrated that, once iPSCs were obtained from these old donors, they have unchanged potential to be differentiated *in vitro* and *in vivo*.

Therefore, even if we can consider that aging may interfere with the onset of the reprogramming process in a cell-autonomous fashion, once the process starts it proceeds to completion until the development of iPSCs.

Contrary to mice, in humans, the aging process does not seem to impair the ability of cells to reprogram. Indeed, several groups generated iPSC lines, which expressed pluripotency markers, were able to differentiate into the three germ layers and to induce teratomas when injected into nude mice, from tissues of older individuals (67, 68), thus demonstrating that reprogramming to iPSC is possible regardless of the donor age. Also, the gene expression profile of these cells confirmed they had been reset to an embryonic-like stage (19, 58, 64, 67).

Lapasset et al. (19) tested the reprogramming feasibility of extreme aging phenotypes, generating iPSCs from post-mitotic cells and fibroblasts isolated from a 101 years old subject. Since then, the generation of iPSC from different centenarian tissue types was obtained (69–71). Interestingly, these iPSC obtained from centenarian fibroblasts were pluripotent and were able to generate all the three embryonic lineages.

The reprogramming process can reverse several age-associated cell features, such as cell-cycle arrest (25), DNA methylation and histone modifications (72), telomere length (73) and expression of pro-inflammatory factors (22).

Indeed, in iPSC the re-expression of telomerase (74), the modification of histone pattern (75) and the improvement of mitochondrial activity with increased energetic output and ROS resistance (76) have been demonstrated.

Hence, reprogramming somatic cells to iPSCs has been shown to reverses their “developmental clock”, restoring it to levels similar to human embryonic stem cells (20). It can be argued that cellular age and identity are not unalterable endpoints, but merely plastic cellular states, mostly depending on the epigenetic code expressed at a given time, whose change is responsible of the whole reprogramming process.

### Cellular Senescence: A Booster for in Vivo Reprogramming

Few studies explored the molecular and cellular contexts affecting *in vivo* reprogramming. Emerging evidence highlight a robust positive association between reprogramming and cellular senescence, which seems to create a tissue microenvironment favoring OSKM-driven reprogramming in neighboring cells.

It is well established that cellular damages associated to reprogramming activate the tumor suppressor genes p53, p21CIP1, p16INK4, and p14ARF, which result in proliferation arrest and, consequently, act as cell-autonomous barriers for cellular reprogramming (22, 24, 58, 76).

Mosteiro et al., in 2016 showed for the first time that the expression of Yamanaka factors *in vivo* not only starts the reprogramming process on some cells but also imposes exacerbated DNA damage on many other cells, leading them toward a senescence state (62).

These reprogramming-induced senescent cells facilitate *in vivo* reprogramming through the paracrine action of some SASP components, in particular, IL-6 (61, 62, 77). Authors demonstrated that OSKM transduction, in the absence of p53, leads to extensive damage and RIS in tissues, resulting in high levels of secreted IL-6 which further enhance reprogramming. On the contrary, in the absence of Ink4a/Arf, RIS is severely compromised, with low IL-6 secretion levels. Although the Ink4a/Arf deficiency should represent a cell-autonomous advantage to reprogramming, the results obtained exhibit a very inefficient reprogramming process, probably due to the *in vivo* absence of RIS and IL-6 secretion (62, 77).

This also might be observed in the reprogramming of aged or injured tissues, where the accumulation of senescent cells may increase OSKM reprogramming by sending signals to surrounding cells. As it has been demonstrated that SASP may also induce somatic stem/progenitor characteristics in several tissues (43), a fascinating picture could be the identification of such a signal capable of inducing tissue regeneration to promote tissue repair.

**FIGURE 1:**
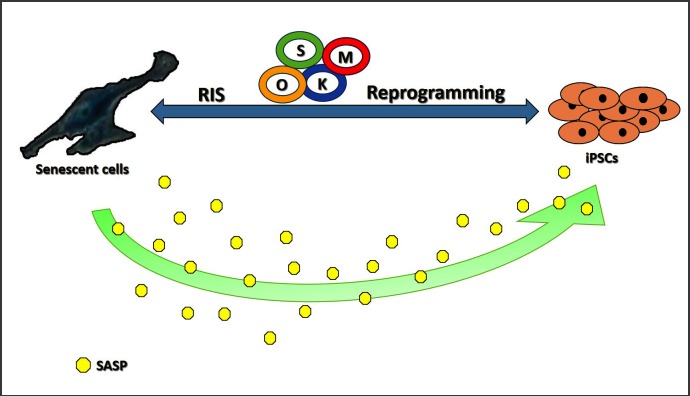
Schematic representation of possible implications of cellular senescence on the reprogramming process. RIS, Reprogramming Induced Senescence; O, Oct4; K, Klf4; S, Sox2; M, c-Myc; SASP, Senescence-Associated Secretory Phenotype.

Possible effects of cellular senescence on the reprogramming process are schematically depicted in Figure 1.

### Cellular Senescence in the Heart

Despite the fact that heart has been traditionally considered a post-mitotic organ, in the recent decades the growth of the cardiovascular research field has led to the hypothesis that the heart can retain a regenerative potential (78), even if limited and still not therapeutically exploitable. Senescence can be described as the irreversible cell-cycle arrest that occurs in mitotic cells; thus, it can seem inappropriate to consider senescence in a context, cardiac, in which cell replication is quite limited. Nevertheless, hallmarks of senescence can be found in the aging heart and recently it has been shown that an increase in senescence markers in the cardiovascular system such as cardiac tissue, great vessels, and pericardium is associated with increased dysfunction and reduced lifespan (79). Thus, in the light of cardiovascular regeneration, cardiac senescence can be considered as organ senescence and analyzed in both cardiomyocytes and non-cardiomyocytes. Cardiac senescence is characterized by the decrease in the number of cardiomyocytes and their increased size, with age and cardiovascular diseases, which represent a fundamental characteristic of cardiac remodeling, along with extracellular matrix increase (80). The numerical changes that occur with aging are mainly due to the age-associated reduced efficiency of autophagy (81).

Telomere length is another hallmark of aging and has been associated with cardiovascular disease development and mortality in elderly patients (82). However, the causality of these associations remains uncertain (83).

Other crucial aspects associated with cardiomyocyte senescence involve age-dependent defects of adrenergic signaling and calcium handling. These two cellular alterations are typically affected by age. Increased norepinephrine circulating levels are the results of reduced plasma clearance and increased spillover from the tissues (82). These alterations lead to the progressive impairment of adrenergic responsiveness, with the consequent β-adrenergic desensitization typically observed in cardiovascular aging. The dysfunction of the myocardial sarcoplasmic reticulum calcium adenosine triphosphatase (SERCA2a) is responsible for the impairment in calcium handling observed in cardiomyocyte aging (84).

Mitochondrial inability to maintain ROS homeostasis contributes to the accumulation of highly reactive products, which increases DNA mutations, inflammation, and cell death pathways activation ultimately leading to cellular senescence (85). Also, the family of nicotinamide adenine dinucleotide-dependent deacetylases termed sirtuins have an established role in human aging (86) and are related to cardiovascular aging and disease development (87).

Non-cardiomyocytes represent the vast majority of cardiac cells population. In particular, cardiac fibroblasts represent the main contributors to the cardiac structure through the production, maintenance, and remodeling of extracellular matrix. Fibroblasts isolated from aged hearts show impaired proliferative capacity and are less responsive to profibrotic stimuli *in vitro* (87). Infarcted aged hearts showed a reduction of collagen deposition in the scar, a delay in the scar stabilization, and reduced yet prolonged inflammation, highlighting a possible age-related cardiac fibroblasts dysfunction (88). In addition to the cellular changes, aging is associated with overexpression of several ECM proteins, such as collagen, fibronectin, and alpha 1 and alpha 5 integrin along with increased collagen cross-linking (89, 90).

### Reprogramming the Aging Heart

Although high cellular plasticity is associated with high regenerative capacity, it also bears high tumorigenicity. Thus, mechanisms aimed at the increase of cellular plasticity may be a double-edged sword. This and other concerns, such as the failure to demonstrate that iPSCs-derived cardiomyocytes, once transplanted, may improve cardiac function, suggest caution in the adoption of such a therapeutic strategy.

Hence, the possibility of remuscularizing an aged or damaged human heart with such approach highlights some critical questions that need to be addressed before *in vitro*-derived cardiomyocyte transplantation can become part of a regenerative therapy.

In recent years it has been developed an alternative method to produce cardiomyocytes *in vitro* as well as *in vivo*, the so-called “direct cardiac reprogramming”. This method bypasses the concerns on plasticity and relative tumorigenicity, as it involves a cell fate switch from a fully differentiated cell type into another, without going through the pluripotent state.

The direct conversion was achieved for the first time in 2010 by forcing expression of tree key lineage-specific factors: Gata4, Mef2c, and Tbx5 (GMT factors) in mice (91).

Since then, different research groups worldwide reported unique combinations of transcription factors, microRNAs and/or chemical compounds capable of engineering mouse and human fibroblasts cell fate to produce cardiomyocyte-like cells both *in vitro* and *in vivo* (92–95 and extensively reviewed in 11). This is a new hope to restore heart function and induce regeneration. Indeed, several elegant efforts have been made to induce heart regeneration by direct reprogramming of cardiac fibroblasts of the infarcted area into induced cardiomyocytes (iCMs). Interestingly, findings revealed that *in situ* trans-differentiation of cardiac fibroblasts into iCMs results in functional improvements in mouse models of MI, yielding more mature cardiomyocytes with more similarity to their endogenous counterparts than in the *in vitro* setting (92, 96–99). These data suggest that cardiac microenvironment may specifically have a positive imp act on the robustness of the process rather than environments of other tissues or *in vitro* conditions.

Interestingly, supporting the positive role of the cardiac milieu, it has been presented that the infarcted adult heart induced more cardiomyocyte maturation and hypertrophy than the neonatal heart (92). This proves that *in vivo* environment of the infarcted ventricle can provide inductive signals specifically for the three-lineage cardiovascular differentiation of iPSCs and maturation of injected cardiomyocytes.

Identification of such powerful inducers in cardiac microenvironment could provide new insights into the mechanisms of cardiac reprogramming. Moreover, other neighboring cell types can make the heart tissue more permissive and favorable to reprogramming.

Indeed, the SASP is characterized by the increase of several soluble factors, either proteins or nonproteins, many of which may play a role in promoting and facilitating tissue reprogramming.

Recently, this topic has become the object of more deep investigations, several of which have led to very interesting yet non-conclusive results.

Recently, intriguing results have been obtained in non-cardiac tissues, such as skeletal muscle and pancreas (61, 62). Mosteiro et al., starting from the evidence that the *in vivo* transduction of OKSM factors induces cellular damage and senescence along with dedifferentiation (62), found a possible role for SASP components in *in vivo* cell reprogramming (77). Also in skeletal muscle, it has been demonstrated that *in-vivo* reprogramming is facilitated by the accumulation of senescent cells (61). These results strongly suggest that cell intrinsic and extrinsic effects of senescence can be pivotal for tissue repair and regeneration, particularly during the aging process and in the presence of tissue damage. Indeed, tissue injury enhances the *in-vivo* lineage reprogramming efficiency in both liver and pancreas and the induction of a senescent program could drive these processes.

With aging, several of the SASP components, particularly those related to proinflammatory signaling, have been shown to be involved in tissue remodeling in both the heart and large arteries. As previously highlighted, a possible interesting role in the remodeling process of the cardiovascular system can be played by IL-6. IL-6, in fact, is significantly increased, secreted and up-regulated in aged Vascular Smooth Muscle Cells (VSMCs) as well as in aged myocardium (100).

To our knowledge, no data on cardiac direct reprogramming and SASP are available.

At cardiac level, in particular, an age-associated increase in myocardial damaged and matrix remodeling seems to be promoted by up-regulation of this cytokine, along with TNF-alpha and both seem to be required for myocardial development (100–102). The role of IL-6, more specifically, seems to be central in the process of myocardial remodeling, either pathological or physiological. In fact, while on the one hand it has recently been showed that deletion of IL-6 attenuates pressure overload-induced left ventricular remodelling, on the other hand, deletion of IL-6 has been demonstrated to be responsible for left ventricular dysfunction, ﬁbrosis, reduced capillary density, and significant alteration of cell populations of the developing and adult heart. Both these pathological and developmental responses to IL-6 signaling occur through the modulation of the activity of STAT3 (103).

IL-6 is also able to enhance *in vitro* reprogramming, by replacing the activity of another IL-6 cytokine family member, the leukemia inhibitory factor (LIF), a related cytokine often used for reprogramming *in vitro* (103, 104).

Given the growing evidence on a possible role of IL-6 in facilitating *in vitro* reprogramming and given the increased levels of this cytokine in the aged heart, it can be argued that also an *in-vivo* direct cardiac reprogramming process could be favorably influenced in the presence of IL-6.

LIF is an essential compound used during the induced cardiomyocytes maturation in the direct cardiac reprogramming process (105). Like IL-6, also LIF acts through a shared gp130 receptor leading to overlapping but characteristic biological actions mediated by the activation of STAT3 (106). Several studies have been performed on the intracellular signals leading to the formation of a “wall of protection” against cardiomyocytes acute stress (104). LIF would seem to have other effects on other cell types in the infarcted myocardium reparative process. In mouse infarcted myocardium, LIF has been shown to contribute to the homing process of bone marrow-derived cardiac progenitors, along with the differentiation of resident cardiac stem cells into endothelial cells (106, 107). Increased circulating LIF in a mouse model of MI not only were protective against cardiomyocyte death but was able to enhance neovascularization and to induce bone marrow cells homing in the heart and their differentiation into cardiomyocytes (108).

## Conclusions

*In vivo* lineage reprogramming-based therapies are being considered to treat a wide range of diseases, and tissue-damage-induced senescence seems to contribute to *in vivo* cellular plasticity via SASP positively. Although this beneficial role for cellular senescence has been demonstrated on muscle and pancreas regeneration, no data are available for a similar effect on cardiac regeneration. Nevertheless, experimental evidence has shown that the reprogramming by GMT in the infarcted adult heart induced more cardiomyocyte maturation and hypertrophy than into the neonatal heart. It may be speculated that this could be ascribed to the induction of a senescence program in the damaged myocardium, thus supporting the positive contribution of the cardiac milieu to the *in vivo* reprogramming process. Hence, it will be of great interest to investigate how specific SASP components would also affect cardiac reprogramming.

## Author Contributions

The authors conceived and wrote the manuscript equally.

## Conflict of Interest Statement

The authors declare that the research was conducted in the absence of any commercial or financial relationships that could be construed as a potential conflict of interest.
